# Muscle histological changes in a large cohort of patients affected with Becker muscular dystrophy

**DOI:** 10.1186/s40478-022-01354-3

**Published:** 2022-04-08

**Authors:** Michela Ripolone, Daniele Velardo, Stefania Mondello, Simona Zanotti, Francesca Magri, Elisa Minuti, Sara Cazzaniga, Francesco Fortunato, Patrizia Ciscato, Francesca Tiberio, Monica Sciacco, Maurizio Moggio, Paolo Bettica, Giacomo P. Comi

**Affiliations:** 1 Fondazione IRCCS Ca′ Granda, Ospedale Maggiore Policlinico, Neuromuscular and Rare Diseases Unit, Milan, Italy; 2 Department of Biomedical and Dental Sciences and Morphofunctional Imaging, University of Messina, Messina, Italy; 3 Fondazione IRCCS Ca’ Granda Ospedale Maggiore Policlinico, Neurology Unit, Milan, Italy; 4 Italfarmaco SpA, Milan, Italy; 5 Dino Ferrari Center, Department of Pathophysiology and Transplantation, University of Milan, Milan, Italy; 6 Department of Surgery, Head and Neck Area, UO Neurosurgery, Fondazione IRCCS Cà Granda Ospedale Maggiore Policlinico, University of Milan, Milan, Italy

**Keywords:** Histology, Becker muscular distrophy, Muscle biopsies, Fibrosis

## Abstract

Becker muscular dystrophy (BMD) is a severe X-linked muscle disease. Age of onset, clinical variability, speed of progression and affected tissues display wide variability, making a clinical trial design for drug development very complex. The histopathological changes in skeletal muscle tissue are central to the pathogenesis, but they have not been thoroughly elucidated yet. Here we analysed muscle biopsies from a large cohort of BMD patients, focusing our attention on the histopathological muscle parameters, as fibrosis, fatty replacement, fibre cross sectional area, necrosis, regenerating fibres, splitting fibres, internalized nuclei and dystrophy evaluation. We correlated histological parameters with both demographic features and clinical functional evaluations. The most interesting results of our study are the accurate quantification of fibroadipose tissue replacement and the identification of some histopathological aspects that well correlate with clinical performances. Through correlation analysis, we divided our patients into three clusters with well-defined histological and clinical features. In conclusion, this is the first study that analyses in detail the histological characteristics of muscle biopsies in a large cohort of BMD patients, correlating them to a functional impairment. The collection of these data help to better understand the histopathological progression of the disease and can be useful to validate any pharmacological trial in which the modification of muscle biopsy is utilized as outcome measure.

**Supplementary Information:**

The online version contains supplementary material available at 10.1186/s40478-022-01354-3.

## Introduction

Skeletal muscle dystrophies are a large and heterogeneous group of inherited disorders characterized by progressive muscle weakness. X-linked Duchenne muscular dystrophy (DMD, OMIM 310,200) and Becker muscular dystrophy (BMD, OMIM 300,376) are among the most severe.

In both disorders, most of the identified mutations are large deletions, spanning one or more exons, the remaining patients harbour exon duplications or less frequently point mutations and small rearrangements [1, 2].

Muscle histopathological changes are central to DMD/BMD pathogenesis. The lack of dystrophin results in sarcolemma instability and increased vulnerability to mechanical stress, causing inflammation, fibre necrosis and fibre regeneration. These changes lead to constant cycles of degeneration and regeneration, but, with age, the repair phase becomes less and less successful as a consequence of exhaustion of satellite cell pools [3]. Muscle fibres are replaced with fat and connective tissue and become progressively weaker (fibroadipose degeneration).

In addition, the mechanisms of muscle repair are defective in both DMD patients and dystrophin-deficient animal models, leading to delayed and or incomplete regeneration [4, 5]. The degree of these mechanisms in the different stages of BMD is less clearly understood. Several histopathologic features—i.e. fibre size variation, centrally nucleated fibres, hypercontracted and regenerating fibres and cellular infiltrates—are common to both DMD and BMD and they may be useful to follow and quantify their progression.

Several of these morphometric parameters have not been thoroughly investigated in a large cohort of BMD skeletal muscle samples nor have they been correlated to the underlying clinical condition. Marked fibre size variability and endomysial fibrosis were observed in studies preceding [6, 7] and following dystrophin discovery [8, 9].

A number of investigational drugs [10] are believed to work by modifying basic muscle parameters including muscle fibre size, amount of total fibrosis, necrosis and regeneration, even if they do not modify the genetic sequence. In our center, 45 BMD patients were recruited to participate to a phase II, randomised, double blind, placebo-controlled study meant to evaluate the micro- and macroscopic effects on skeletal muscles of the histone deacetylase (HDAC) inhibitor Givinostat as previously described [11].

Our aims were to examine and describe, at baseline condition, namely before pharmacological treatment, the histopathological features of skeletal muscles in a large cohort of ambulant BMD patients and to identify possible correlations between the clinical disease stage and muscle histological changes. This study provides the opportunity to examine the disease-associated muscle histopathology in a relatively large cross-sectional sample, thereby providing relevant information about the histopathological progression of the disease that could become useful biomarkers for future studies aimed at evaluating the effect of any therapy on muscle tissue.

## Material and methods

### Patients

This study was conducted on forty-five adult males (aged 19–65 years) affected by BMD. Familial cases included two brothers (pts 14 and 15) and two twin brothers (pts 28 and 29).

Genetic diagnosis included both in-frame exon deletions/duplications and missense mutations in dystrophin gene, as showed in Table 1. All patients, with a cardiac ejection fraction of not less than 50%, were able to walk between 200 and 450 m in a 6-min walk test (6MWT). Regular performance evaluations were Motor Function Measure (MFM) test, 6MWT, muscle strength measured by hand-held myometry and the following timed-function tests: rise from floor, 10 m run/walk test, climb four standard steps.

**Table 1:** Characteristics of patients with BMD

	BMD (n = 45)
Age (years)	38.60 (11.42)
Range	19–62
Age at Onset (years)	14.85 (10.79)
Range	2–40
Disease duration	23 (19–29)
Range	6–58
BMI, mean (SD)	23.28 (4.22)
Mutations	
Deletions 45-x	29 (64.44%)
Mutations proximal to exon 45	13 (28.89%)
Deletions distal to exon 45	3 (6.67%)
Treatment ^(*n=41)^	
Steroid therapy	3 (7.32%)
MFMD1	19.11 (4.82)
MFMD2	35.27 (1.3)
MFMD3	20.69 (0.6)
MFMD Total	75.04 (5.86)
6MWT (m)	361 (71.11)
4-Stair Climb Score ^(*n=43)^	5 (2–5)
10 m Score	4 (4–4)
Gowers Score ^(*n=39)^	2 (2–3)
Right Biceps Brachial Strength	68 (36.50–136.5)
Left Biceps Brachial Strength	74 (39–130.5)
FE%	61 (55–64)

Data are given as mean (SD), Number (percentage), or median (interquartile range)

BMD: Becker Muscular Dystrophy; yrs: years; BMI: Body Mass Index; SD: Standard Deviation; MFMD Motor Function Measure Domain; 6MWT: 6 Minute Walking Test; 10mScore: 10 meters score; FE%: Fraction Ejection percentage

All BMD patients underwent a biceps brachii skeletal muscle biopsy.

In order to compare morphometric data, fifteen biceps brachii muscle biopsies previously performed for clinical reasons in age-matched male individuals deemed to be free of muscle disorders, were used as controls.

All participants provided written informed consent. Study protocol and consent forms were approved by the local Ethics Committee.

Table 1 shows the demographic and clinical characteristics of study participants.

### Histological analysis and morphometry

Muscle tissue specimens were frozen in isopentane precooled in liquid nitrogen and stored in liquid nitrogen pending use.

For histological analysis, 8 µm-thick cryosections were picked and processed for routine staining with haematoxylin and eosin (H&E) and myosin ATPase (pH 9.4). On each section, four randomly, non-overlapping selected fields were photographed at 20× magnification, using optical microscope Leica DC200 equipped with camera and IM50 image analysis software (Leica Microsystems, Wetzlar, Germany). All morphometric analyses were performed using ImageJ 1.51j8 (https://imagej.nih.gov/ij/download.html) and LAS 4.9.0 (Leica Application Suite) software.

The following morphological parameters were evaluated on H&E-stained muscle sections: muscle fibre area (MFA) fraction, total fibrosis (both perimysial and endomysial) and fatty replacement.

MFA was obtained including all fibres, both intact and partial (localized at the edges of the field) in each microscopic field. We also considered histological structures occupying the analysed field, namely vessels and peripheral nerves.

On the same H&E muscle sections, centronucleated fibres, degenerating/necrotic fibres and splitting fibres were counted. All measures were expressed as percentage of the total area for each field analysed. Following a previously reported protocol [12], two operators blindly evaluated each muscle biopsy. Fibre size variability, inflammatory infiltration, fibre hypertrophy/atrophy, angulated fibres, fibres grouping was evaluated by two operators according to their extent and/or severity [13].

On myosin ATPase-stained section, cross sectional area (CSA) of type I and type II fibres was measured. CSA was assessed by manually drawing the perimeter of each fibre and by calculating the corresponding area (μm^2^) using LAS version 4.9.0 software. Only transverse fibres were included in the analysis.

### Evaluation of regenerating fibres

Quantification of regenerating fibres was manually performed on four muscle sections after double immunohistochemistry for fetal myosin (anti-MYH3) and for the sarcolemma protein caveolin-3 (CAV-3) to delineate individual muscle fibres.

Briefly, each muscle section (7 μm thick) was fixed with ice-cold acetone (2–3 min), washed in phosphate buffer (PBS) and incubated for 30 min with 5% bovine serum albumin (BSA). Slides were incubated with primary rabbit polyclonal antibody MYH3 (1:200; Sigma Aldrich) and mouse monoclonal antibody CAV-3 (1:100; BD Transduction Lab, San Jose, CA, USA) for 2 h, followed by 1 h with the appropriate secondary antibody conjugated to AlexaFluor-488 (1:500; Molecular Probes, Carlsbad, CA, USA) or rhodamine dye (1:600; Merck Millipore, Burlington, MA, USA). Finally, after PBS washes, slides were mounted with anti-fading reagent Fluormount (Thermo Fisher Scientific, Waltham, MA, USA). As control, sections were incubated either with isotype specific IgG, or the primary antibody was omitted.

Four randomly non-overlapping selected fields on each section were photographed at 20× magnification. In each field both MYH3 positive fibres and the total number of fibres were counted using ImageJ 1.51j8.

### Western blot analysis

For immunodetection of endogenous dystrophin, a small fragment of muscle biopsy was homogenized in extraction buffer (4% SDS, urea 4 M). Lysate protein was loaded on 6% polyacrylamide-SDS gel and transferred to a nitrocellulose membrane (Schleicher and Schuell, Keene, Nh, USA). Membranes were probed with the following primary antibodies: dystrophin Rod domain mouse monoclonal antibody (NCL-DYS1, dil. 1:200), dystrophin C-terminus mouse monoclonal antibody (NCL-DYS2, dil. 1:80) all from Novocastra Laboratories (Newcastle upon Tyne, UK).

Actinin (monoclonal antibody, 1:6000 Sigma Aldrich) was used as indicator of protein loaded [14]. The membranes were incubated with rhodamine or fluorescein goat anti mouse secondary antibodies (LI-COR, Lincoln, NE, USA).

Immunoreactive bands were visualized by ODYSSEY LI-COR Model 2800 and quantitated densitometrically using Image J 1.46r software. Dystrophin bands were normalized to actinin band and expressed as percentage with respect to control values.

### Statistical analysis

No formal calculation of the sample size was done as analyses were done retrospectively and included all BMD adults who fulfilled the inclusion criteria. Baseline characteristics were summarized using standard descriptive statistics. Continuous variables were described as mean (SD) or median (IQR), as appropriate, and categorical data were summarized as absolute frequencies and percentages.

We compared groups using Mann–Whitney tests (two groups) and Kruskal–Wallis tests with post-hoc Dunn’s test (three groups). The association between categorical variables was evaluated using the Fisher’s exact test. Correlations between quantitative variables were visualized by scatterplots and quantified using Spearman’s rank correlation coefficients. We explored adjusting for multiple testing (false discovery rate).

Multivariate analysis methods were used to identify relevant patient clusters and variables responsible for class discrimination [15, 16]. We first used principal component analysis (PCA) to lower the dimensionality of the data and to identify distinct clusters and potential outliers within the data sets. Outliers were identified using score plots in combination with Hotelling’s T2 and distance to model in X-space (DModX) [17, 18]. In addition, in order to maximize identification of relevant subgroups of patients (subtle clusters), a bottom-up hierarchical clustering analysis (HCA) was applied to the principal component score vectors using the default Ward linkage criterion. Clusters were identified based on the resulting dendrogram and partial least squares-discriminant analysis (PLS-DA) was performed using group membership as Y-variables and patient data as predictors (X-variables). The PLS-DA model was computed to identify associations between the X-variables and the groups, as visualized on the corresponding loading plot. Traditional statistical analysis was performed using R (http://www.r-project.org, version 3.5.1) in RStudio (http://www.rstudio.com, version 1.1.456), and SIMCA® 16 Software (Umetrics AB, Umeå, Sweden) was used for multivariate data analysis. P-values *(*p* < 0.05), **(*p* < 0.01) or ***(*p* < 0.001) were considered significant.

## Results

### Description of population

We studied 45 patients with BMD. The average patient age was 38.6 years (range 19–62 years), the average age of symptoms onset was 14.85 years (range-2–40 years). The median disease duration was 23 years (IQR 19–29). Demographic characteristics are summarized in Table 1. All patients underwent a muscle biopsy. In addition, fifteen age-matched healthy controls who fulfilled the criteria and had already undergone muscle biopsy were included in this study.

### Clinical assessment

Clinical evaluation of BMD patients is summarized in Table 1, motor function was assessed by 6MWT, 4-Stair Climb Score, 10 m Score, Gowers Score and MFM test, whereas biceps brachii muscle strength was measured by hand-held myometry.

Mean value for 6MWT was 361 ± 71.11 m and mean scores for 4-stair climb, 10 m and Gower’s were 5 (with 2 missing), 4 and 2 (with 6 missing) respectively. Mean values for right and left biceps brachii strength were 68 and 74 N respectively.

The mean value of total MFM obtained from our BMD patients was 75.04 ± 5.86. The three dimensions of MFM scale showed a mean value of 19.11 ± 4.82 for the first factor D1 containing 13 items testing standing position and transfers, a mean value of 35.27 ± 1.3 for the second factor D2 consisting of 12 items evaluating axial and proximal limb motor function, and a mean value of 20.69 ± 0.6 for the third factor D3 containing 7 items evaluating distal motor function.

### Muscle fibre area, fibro-fatty tissue substitution and cross-sectional area variability

All 15 muscle biopsies from control patients showed a regular muscle architecture with homogeneous fibre size, no/minimal (< 2%) necrotic/regenerating muscle fibres, internalized nuclei and/or splitting fibres. The tissue samples consisted of over 85% skeletal muscle, and ~ 10% fibrosis/connective tissue. No fat tissue replacement was detected.

Muscle biopsies from BMD patients showed marked variability, ranging from an almost normal morphology to a severe dystrophic pattern. Histological data analysis on muscle fibre area, total fibrosis/connective tissue and fatty replacement showed that overall, in BMD, MFA percentage was significantly reduced (76.84%) compared to controls (90.85%, *p* = 0.0001), along with a significant increase in percentage of fibrotic tissue (22.45% in BMD compared to 9.15% in controls, *p* = 0.0001). Fat tissue replacement was found in a significant number (*n* = 9) of BMD patients, but presence of “other tissue” did not differ between patient and control populations. (Table 2).

**Table 2:** Histological characteristics of BMD patients and controls

	Study participants		
	BMD (n = 45)	Controls (n = 15)	Adjusted P value ^a^
CSA I (µm^2^) Fibre size	4074 (2852–5833)	3991 (3387–4695)	1
CSA I Fibre size variability	3316 (2246–4799)	1200 (814–1463)	**0.0001**
CSA II (µm^2^) Fibre size	4680 (2649–5978)	4681 (3333–5604)	1
CSA II Fibre size variability	4511 (3733–6506)	1623 (1241–2231)	**0.0001**
Nuclear Centralizations %	4.5 (3–5)	1 (0–1.5)	**0.0001**
Regenerating fibres %	3.4 (0–6.82)	0 (0–0)	**0.0001**
MFA %	76.84 (62.68–83.10)	90.85 (88.91–92.01)	**0.0001**
Fatty tissue %	0 (0–0)	0 (0–0)	**0.0001**
Range	0–16.52	0–0.001	
Fibrotic tissue %	22.45 (15.89–34.70)	9.15 (7.72–10.76)	**0.0001**
Other tissue %	0 (0–0)	0 (0–0)	0.39
Range	0–3.3	0–0.24	
Splitting fibres %	3.1 (1.03–5.74)	0 (0–0)	**0.0001**
Necrosis fibres %	0.79 (0–1.54)	0 (0–0)	**0.0052**

Statistically significant *p* values are shown in bold

Data are given as median (interquartile range). a Mann–Whitney U test

BMD: Becker Muscular Dystrophy; CSA: cross sectional area; MFA: muscle fibre area

The evaluation of other histological features showed that necrotic (*p* = 0.0052) and splitting (*p* = 0.0001) fibres were significantly higher in BMD patients than in controls (Table 2).

The quantification of fibres with nuclear centralizations highlighted a significant increase in BMD patients compared to controls (Table 2). H&E-stained muscle sections showed, in addition, a minimal presence of inflammatory cell infiltrates in 23 patients, no cell infiltrates were detected in 19 patients and only in 1 patient a marked presence of inflammatory cell infiltrates was detected.

Type I and II fibres cross sectional area (CSA) size variability was markedly higher overall in BMD (~ 3 times) compared to controls (*p* = 0.0001) while no significant difference was observed for mean fibre size (Table 2).

The average number of MYH3-staining positive fibres was significantly different between BMD and controls (*p* = 0.0001) (Table. 2).

Data obtained from quantification of MFA and fibroadipose substitution suggested a classification of patients into three groups characterized by different degrees of tissue impairment: mild, moderate, and severe.

**Fig. 1:**
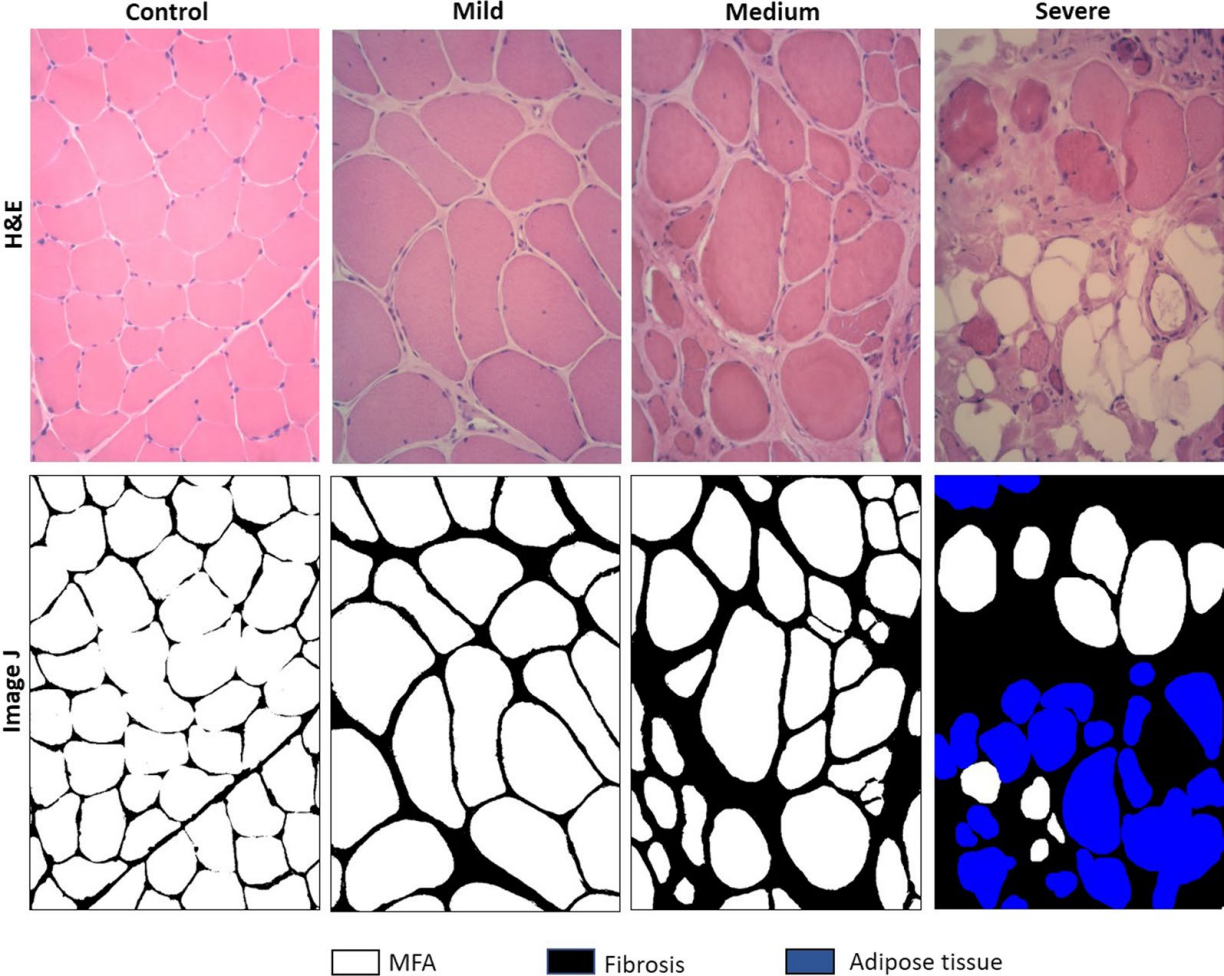
Connective and adipose tissue assessment. Haematoxylin & Eosin staining (top panel) and color subtraction using Image J 1.51j8 software (bottom panel) to measure total fibrosis. Magnification ×20. **a** control muscle, **b** representative patient of cluster 1, **c** cluster 2 and **d** cluster 3. White color corresponds to MFA, black to fibrosis and blue to adipose tissue

A representative H&E picture and the corresponding color subtraction reworked ImageJ image from each picture, is depicted in Fig. 1.

**Fig. 2:**
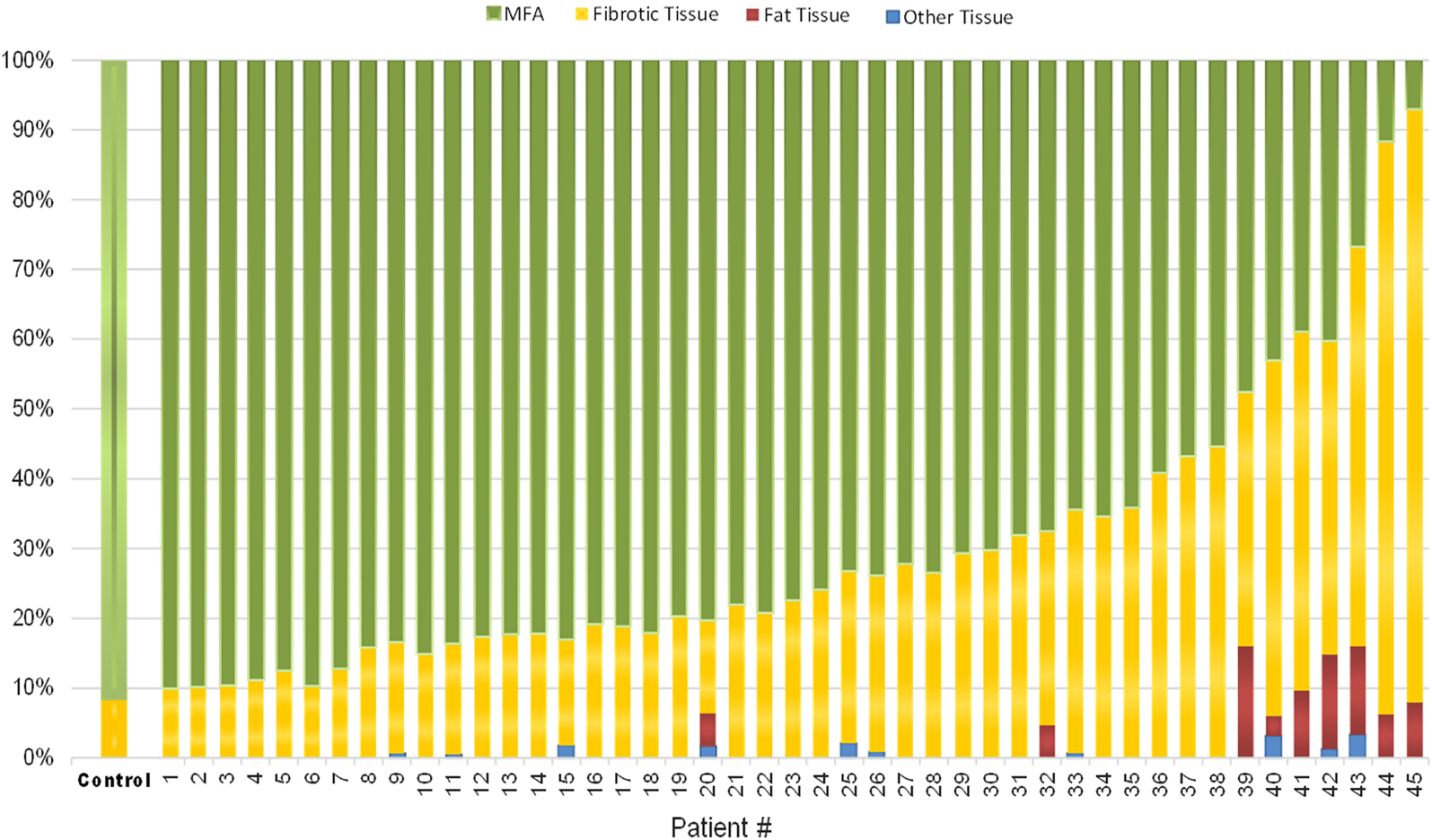
Stacked bar graph of the different tissue components measured in muscle samples of controls and BMD patients. The first column represents the control group (*n* = 15 total), each other column represents an individual patient. The height of each color indicates the relative abundance of a specific tissue component, as indicated in the legend on the top (i.e., Green color indicates the percentage of MFA, yellow the connective tissue, red the adipose tissue and blue the other histological structures). On the left are displayed patients with mild changes in muscle biopsy, on the right patients with a severe fibroadipose replacement

Stacked bar graph in Fig. 2 shows the different percentage distribution of MFA, fibroadipose or other tissue in each patient.

### Western blot

Western blot analysis was performed on 39 patients (6 patients were excluded because muscle homogenate was insufficient for bands quantification). Results obtained using antibodies for both rod and C-terminus domains showed a similar behaviour characterized by a great variability in residual dystrophin protein quantity ranging from 10 to 78% compared to controls (Additional file 1: Table S1).

### Correlation analysis

Correlation analysis of histological characteristics showed that histological parameters did not correlate with genetic mutations, steroid treatment or BMI. In a subgroup analysis including patients in whom Western blot data were available, no correlations with histological characteristics were found.

Moreover, we identified negative correlations, specifically between disease duration and splitting fibres (r = − 0.47, P = 0.001) and between age and regenerative fibres (r = − 0.33, P = 0.03).

**Fig. 3:**
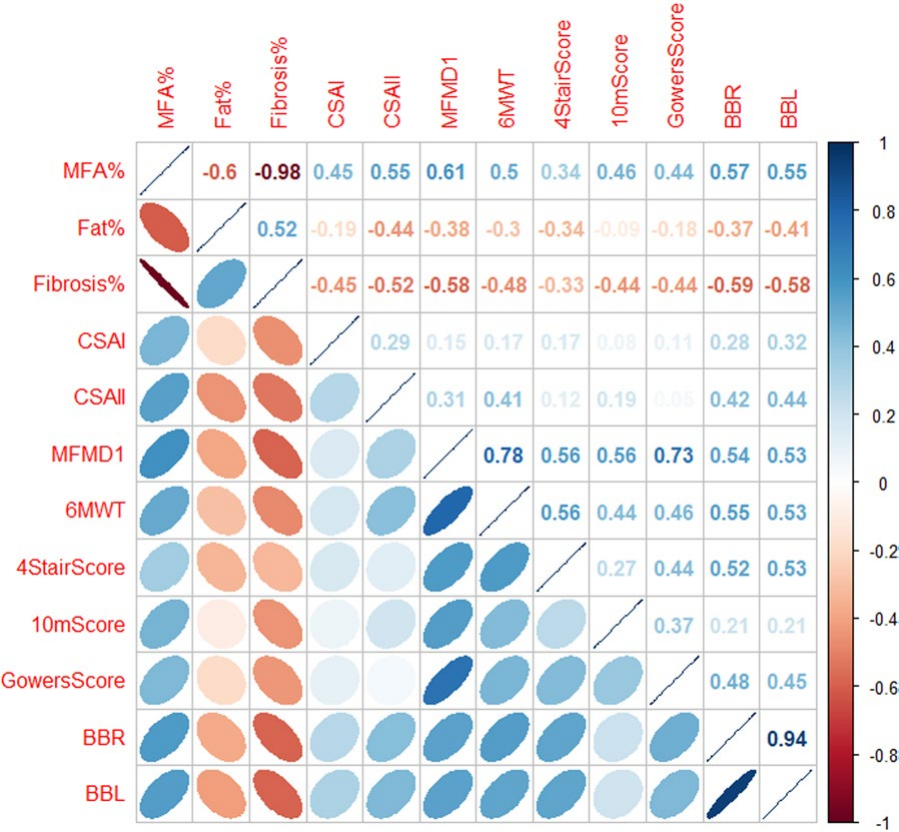
Correlation plot displaying correlations between histological and clinical parameters in BMD patients**.** Positive correlations are displayed in blue and negative correlations in red. Color intensity and the eccentricity of the ellipse are indicative of the strength of correlation. On the right side of the correlogram, the color legend shows the correlation coefficients and the corresponding colors

The correlation analysis between histological parameters and clinical outcomes showed that in BMD patients the percentage of MFA positively correlated with most of the clinical outcomes, more for MFMD1 (r = 0.61), RBB (right brachial biceps strength) (r = 0.57), LBB (left brachial biceps strength) (r = 0.55), 6MWT (r = 0.5) and less for 10 m score (r = 0.46) and Gowers score (r = 0.44). Furthermore, the percentage of MFA positively correlated with CSA II size (r = 0.55), less with CSA I (r = 0.45) and negatively with fibrosis (r = 0.98). The most interesting correlations are shown in the correlation plot (Fig. 3).

### Multivariate data analysis

**Fig. 4:**
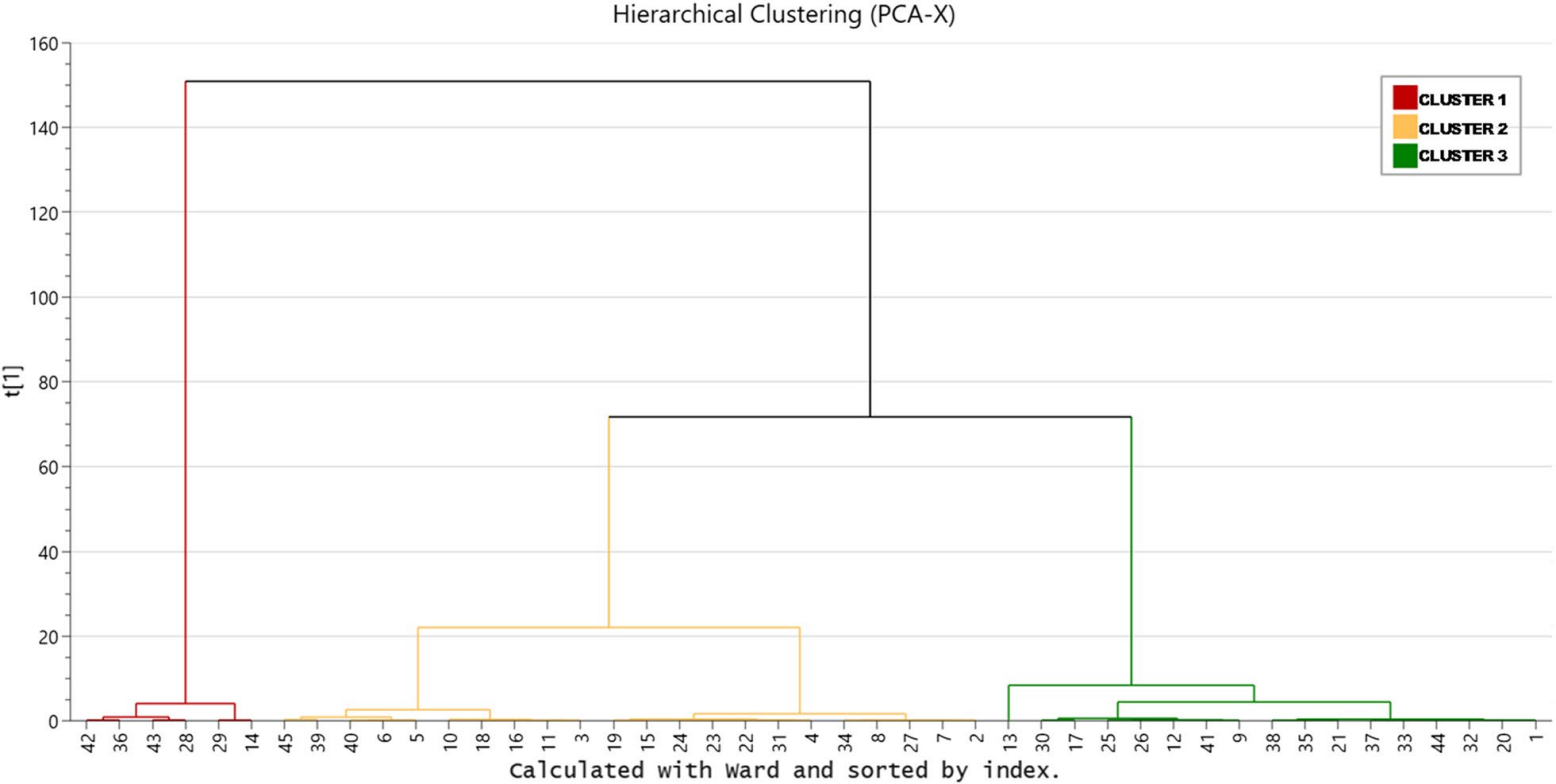
Dendrogram representing three clusters in the hierarchical cluster analysis (HCA). The vertical scale (Y-axis) is a similarity/dissimilarity measure. The individual observations (patients) are on the bottom row (X-axis)

PCA analysis identified no influential outliers, consequently, all 45 patients were retained for subsequent analyses. The resulting PCA model (1 PC, R2 = 0.24, Q2 = 0.12) (Additional file 2: Fig. S1 Bidimensional score plot) was used for performing the HCA. In the resulting dendrogram, a level of three clusters was chosen for subsequent analyses (Fig. 4): cluster 1 (n = 6, 13%); cluster 2 (*n* = 22, 49%) and cluster 3 (*n* = 17, 38%).

**Fig. 5:**
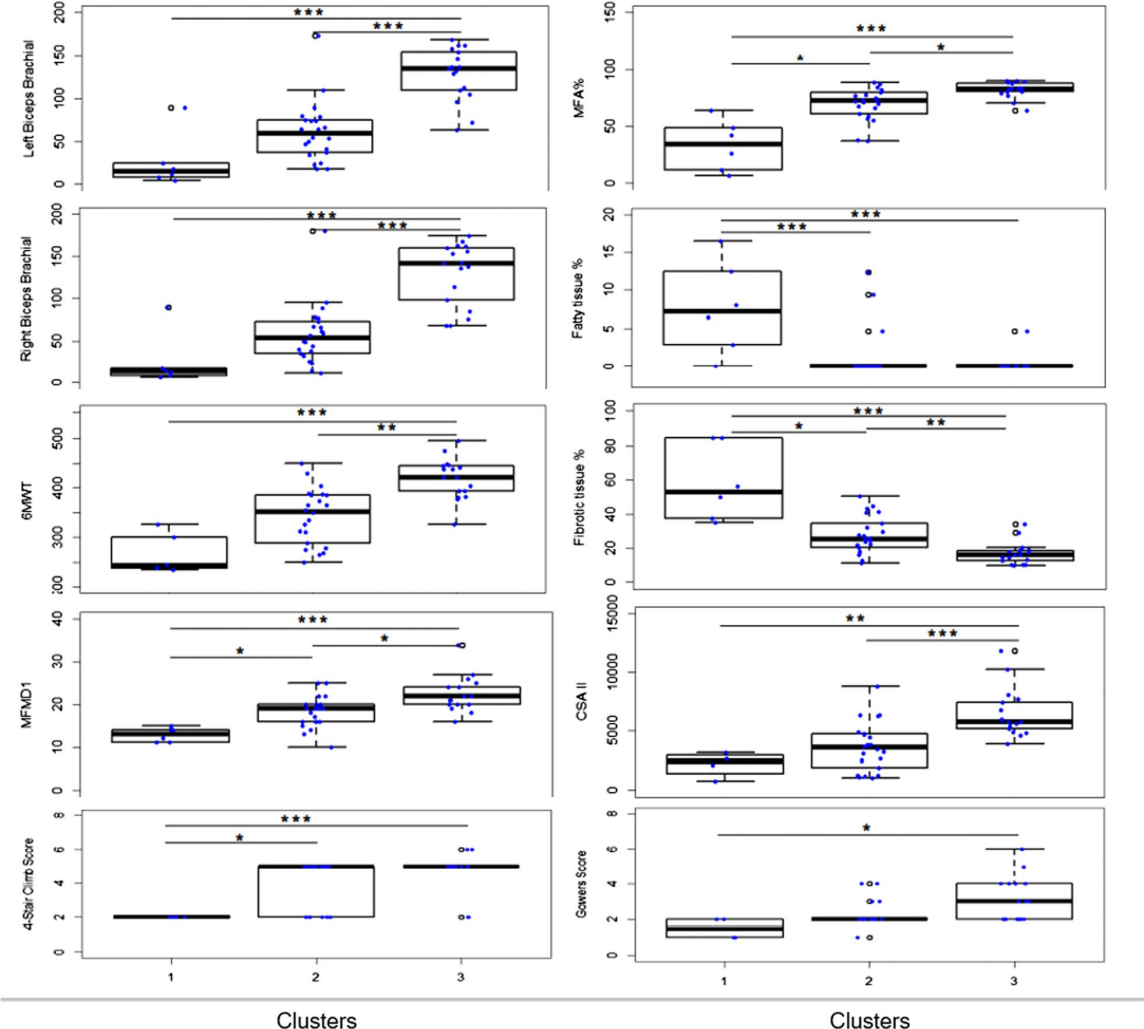
Group differences for the relevant variables. The black horizontal line in each box represents the median, with the boxes representing the interquartile range (Tukey-style whiskers). Each individual value is plotted as a dot superimposed on the graph. *(*p* < 0.05), **(*p* < 0.01) or ***(*p* < 0.001)

Based on HCA, a PLS-DA model (R2 = 0.17, Q2 = 0.39, *p* < 0.001 by CV-ANOVA) (the 3 clusters are shown in Additional file 3: Fig S2 score plot 3D) and the corresponding loading plot were obtained to determine how demographic, histological or clinical parameters relate to each other as well as to the cluster belonging. The findings of the loading plot (Additional file 4: Fig. S3) can be summarized as follows: cluster 1 was characterized by a high percentage of adipose and fibrotic tissue and low MFA and bad performances in functional tests. Cluster 3 was characterized by low fat and fibrotic tissue, high MFA and good performances in functional tests. Cluster 2 was in many ways like cluster 3 but with less MFA and more fibrotic tissue. We also evaluated the differences among the clusters using traditional statistical testing. Values are reported in Table 3 and Fig. 5 illustrates important group differences for relevant variables.

**Table 3:** Characteristics of the three clusters

Variable	Cluster 1 (n = 6)	Cluster 2 (n = 22)	Cluster 3 (n = 17)	P-value
Age	28 (26–38)	42.5 (26.75–48)	38 (30.5–43.5)	0.29
Mutation				**0.03**
Deletions 45-x	1 (17)	17 (77.27)	11 (64.44)	
Deletions distal to exon 45	0 (0)	1 (4.55)	2 (611.76)	
Mutations proximal to exon 45	5 (83)	4 (18.18)	4 (23.53)	
BMI	21.40 (30.36–22.61)	22.10 (20.11–24.85)	24.02 (21.36–28.48)	0.2
Disease duration	25 (23.5–26.5)	22 (9.75–30.25)	23 (20–28.5)	0.77
MFMD1	13 (11–14.25)	19 (16–20)	22 (20–24.5)	** < 0.0001**
6MWT	242 (237–306)	352 (286–386)	420 (388–446)	** < 0.0001**
4-Stair climb score ^(* n=42)^				
2	5 (100)	6 (28.57)	2 (11.76)	**0.001**
5	0 (0)	15 (71.43)	13 (76.47)	
6	0 (0)	0 (0)	2 (11.76)	
10 m score				0.21
3	1 (16.67)	2 (9.09)	0 (0)	
4	5 (83.33)	19 (86.36)	12 (70.59)	
5	0 (0)	1 (4.55)	3 (17.65)	
6	0 (0)	0 (0)	2 (11.76)	
Gowers Score ^(* n=38)^				0.17
1	2 (50)	1 (5.56)	0 (0)	
2	2 (50)	13 (72.22)	8 (47.06)	
3	0 (0)	2 (11.11)	3 (17.65)	
4	0 (0)	2 (11.11)	4 (23.53)	
5	0 (0)	0 (0)	1 (5.88)	
6	0 (0)	0 (0)	1 (5.88)	
Right Biceps Brachial Strength	12.67 (7.75–34.58)	53.17 (34.50–73.67)	141.3 (91.33–160.7)	** < 0.0001**
Left Biceps Brachial Strength	15 (7.08–40.25)	59.33 (36–75.5)	135 (107–155.8)	** < 0.0001**
Nuclear Centralizations	4 (2–5)	5 (3–7)	4 (3–5)	0.25
MFA %	34.18 (10.73–52.82)	72.55 (60.89–79.86)	82.59 (79.96–88.25)	** < 0.0001**
Fibrotic tissue %	53.05 (36.79–84.78)	25.40 (19.69–36.06)	15.90 (11.57–18.97)	** < 0.0001**
Fatty tissue %	7.22 (2.12–13.49)	0 (0–0)	0 (0–0)	** < 0.0001**
Other tissue %	0.34 (0–3.14)	0 (0–0)	0 (0–0.25)	0.12
CSA I	3007 (1711–4780)	3334 (2250–6006)	5234 (4023–6390)	0.07
CSA I Fibre size variability	4667 (3519–5254)	2668 (2018–4596)	3691 (2509–4651)	0.17
CSA II	2382 (1093–3042)	3555 (1720–4776)	5769 (5044–7574)	** < 0.0001**
CSA II Fibre size variability	3868 (1528–4550)	4422 (3162–6079)	4726 (4067–7309)	0.17
Regenerating fibres %	0.61 (0.12–12.94)	4.18 (0–7.34)	3.4 (0.3–6.11)	0.88
Splitting fibres%	2.17 (0–4.17)	4.6 (2.27–6.71)	1.84 (0.83–5.5)	0.07
Necrosis fibres%	1.45 (0.66–1.61)	0.81 (0–2.19)	0 (0–1.09)	0.12

Statistically significant *p* values are shown in bold

Data are expressed as median (25th-75th percentiles) and number (frequencies), as appropriate. Statistics computed by Kruskal Wallis Test, except for categorical variables (Fisher’s exact test)

BMI: Body Mass Index; MFMD Motor Function Measure Domain; 6MWT: 6 Minute Walking Test; 10mScore: 10 m score; MFA: muscle fibre area; CSA: cross sectional area

### Histological parameters

MFA, fat and connective tissue percentages differed substantially among the three clusters. In detail, Cluster 1 was characterized by a statistically lower MFA, less than half compared to the other clusters, and higher fibrosis (Table 3 and Fig. 5). Notably, the greatest difference concerned adipose tissue (median 7,22 in Cluster 1 vs 0 in the other clusters).

CSA II size also differed statistically among the three clusters (*p* < 0.0001). No other differences were found (Table 3, Fig. 5).

### Clinical parameters/scales

Significant differences were detected among clusters on the MFMD1 test (*p* < 0.0001), the 6MWT (*p* < 0.0001), the 4-stair climb score (*p* = 0.001), right and left biceps brachii strength (*p* < 0.0001), while there were no differences concerning 10 m and Gowers scores (Table 3).

## Discussion

In the present study, we examined skeletal muscle biopsies from 45 ambulant BMD patients with an average age of 38 years and a disease duration of 23 years, with the aim to describe their histological features and to identify possible correlations with clinical evolution.

In BMD patients skeletal muscle histology is extremely variable. More severely affected muscles may resemble DMD tissues, whereas in less-affected cases the pathology may be limited to variation in fibre size and mild fibrosis, with varying degrees of degeneration or necrosis.

The first two studies reporting histological features in a large number of BMD patients date back to 1978 [6] and 1984 [7]. The authors described a histological pattern characterized by several myopathic changes, namely marked variation in fibre size with fibre splitting and endomysial fibrosis, associated with neuropathic alterations as group atrophy, pyknotic nuclear clumps and angulated fibres.

In 1991, Kaido and colleagues [8] analysed muscle biopsies from 20 BMD patients with confirmed dystrophin defects. Results highlighted absence of correlation between histological characteristics and dystrophin abnormality, but histological differences segregated based on age at biopsy. In younger patients, necrosis and regeneration were prevalent, conversely, chronic myopathic changes were observed in older patients.

Data obtained from our study are no exception: BMD muscle biopsies showed marked histological variability, ranging from an almost normal morphology to a severe dystrophic pattern with a marked fibroadipose replacement. As a confirmation of this great clinical variability, in our cohort we found marked differences in both histological and clinical traits also in two brothers and two twin brothers.

In accordance with the well documented difficulty to correlate histological and clinical characteristics with genotype in BMD patients [19], our histological data do not correlate with genetic mutations. The most interesting result of our study is the accurate quantification of MFA and of connective tissue in each muscle biopsy.

The first comparison made between BMD patients and controls showed a significant reduction of MFA with a concomitant significant increase of fibrotic tissue in BMD compared to controls. The number of regenerating fibres, evaluated for their central role in muscle repair, is significantly increased in BMD compared to controls. Similarly, as a consequence of degeneration and regeneration processes, both necrotic and splitting fibres are significantly higher in BMD patients than in controls. Western blot analysis showed highly variable residual levels of dystrophin from one patient to another, in agreement with already reported data [20]. The percentage of dystrophin content in patients’ muscle does not always well correlate with clinical findings given also the role in the progression of pathology of genetic modifiers as SPP1, CD40 and LTBP4, originally identified in DMD patients and then observed also in BMD [21]. Genetic modifiers are especially involved in TGF-β1 fibrosis and inflammation pathways that are activated during fibro-fatty deposition process, the most relevant histopathological alteration that caused progressive loss of normal muscle tissue architecture and consequent progressive impairment in muscle contraction [22].

The in-depth statistical analysis conducted allowed to finely divide our cohort of BMD patients into three clusters classified, according to the clinical and histological features, in mild, moderate and severe. This classification correlates neither with age, nor with genetic mutation or disease duration. The histological analysis showed significant differences above all for MFA and for fibrosis. The important deposition of fibrotic tissue progressively replaced muscle fibres, altering the normal muscle architecture, compromising contractile function, preventing fibre regeneration and ultimately obstructing the targeting of specific therapies. [12, 23, 24].

The severe cluster is characterized by significant increase in deposition of both connective (53.05%) and adipose tissue (7.22%) with a consequent marked reduction of MFA (34.18%). The moderate and mild clusters had 25.40% and 15.90% fibrotic tissue respectively and no fatty infiltration was detected. In these two clusters, the residual MFA, 72.55% and 82.59% respectively, still allowed a certain degree of muscle residual functionality. Patients belonging to the severe cluster tended to have a lower number of regenerating fibres compared to the other clusters, though variability was high (IQR 0.12–12.94). This along the relatively modest percentage of splitting fibers may suggest that the regenerating process is unable to keep pace with requirement for new fibres. Future studies are required to determine the clinical value of these initial findings.

As a consequence of marked fibrosis, a deterioration of muscle contractile function appeared, which may explain the worst performance in clinical test applied (biceps muscle strength, 6MWT, 4-stair climb and MFMD1) in more severe patients.

In the last two decades, the clinical environment of dystrophinopathies has seen impressive advancements in the field of new molecular therapies. Most strategies aim at the molecular correction of dystrophin deficiency in selected series of patients with specific mutations [25, 26]. This approach showed beneficial results in DMD patients, but not in BMD patients, in which most of the molecular defects concern the quantity and quality of dystrophin and are not amenable to correction [26].

This clinical heterogeneity makes clinical trial design for drug development quite complex. In this regard, it is essential to gather data on the natural history of BMD patients, and muscle biopsy is still a useful diagnostic tool [27], even if the advances in molecular diagnostics have diminished its role.

Data obtained from our statistical analysis suggested the importance of selected suitable clinical tests to be applied during pharmacological treatment or clinical trials, to be able to efficiently monitor patients’ clinical progress.

It is important to underline that this study recruited patients participating to a clinical trial and therefore meeting specific inclusion criteria. In details, ambulant BMD patients aged ≥ 18 to ≤ 65 years and able to perform 6MWT at screening with a minimum distance of 200 m and maximum distance of 450 m, were recruited. These inclusion criteria prevented us from examining muscle biopsies from younger and older patients, therefore, individuals with a very mild or otherwise very severe disease were excluded.

## Conclusion

At present, this work has collected one of the largest cohorts of ambulant BMD patients, providing relevant information about histological picture and showing extremely significant correlations between histological traits and some functional data making this information useful for any pharmacological trial in which the modification of muscle biopsy is utilized as outcome measures.

## Supplementary Information

**Additional file 1** Western Blot Analysis

**Additional file 2** Bidimensional score plot of the principal component analysis (PCA) applied to the data set of the 45 BMD patients (each dot represents a patient)

**Additional file 3** Three-dimensional score plot of the PLS-DA model showing the three clusters in which BMD patients are classified according to different histological and clinical traits. Each dot represents a patient. Green dots identified patients of cluster 1, blue dots of cluster 2 and red dots of cluster 3.The axis score t[1] represents the latent variable of the model. The latent variable is a mathematical construct that ‘summarizes’ the variables registered in the study. PLS-DA: partial least squaresdiscriminant analysis.

**Additional file 4** Loading plot of the PLS-DA model. The loading plot is complementary to the score plot and summarizes how the X-variables relate to each other as well as to group belonging (Y-variable symbolized by a group dot). X-variables located near a group dot are positively associated with that group. PLS-DA: partial least squares-discriminant analysis
