# Supplementary material for: Muscle histological changes in a large cohort of patients affected with Becker muscular dystrophy
Source: Acta Neuropathol Commun. 2022 Apr 8;10:48. doi: 10.1186/s40478-022-01354-3 (PMC8994373; doi:10.1186/s40478-022-01354-3)
Supplement: Supplementary file 1 — Additional file 1 Western Blot Analysis [file 40478_2022_1354_MOESM1_ESM.docx]

**Table S1:** Western Blot Analysis.

| ID Patient | WB Dys1 | WB Dys2 |
| --- | --- | --- |
| 1 | 52,37 | 19,41 |
| 2 | 45,97 | 28,16 |
| 3 | 16,55 | 12,28 |
| 4 | 49,28 | 27,81 |
| 5 | 68,74 | 11,13 |
| 6 | 44,17 | 21,00 |
| 7 | 19,94 | 15,25 |
| 8 | 33,63 | 20,00 |
| 9 | 16,78 | NV |
| 10 | 20,75 | NV |
| 11 | 36,64 | 21,93 |
| 12 | 18,45 | 6,31 |
| 13 | 22,78 | 13,25 |
| 14 | 32,17 | 20,34 |
| 15 | 32,12 | 22,11 |
| 16 | 40,00 | 20,00 |
| 17 | 22,84 | 13,68 |
| 18 | NV | NV |
| 19 | 27,78 | 12,12 |
| 20 | NV | 22,28 |
| 21 | 9,92 | NV |
| 22 | 23,16 | 9,34 |
| 23 | 37,42 | NV |
| 24 | 42,07 | 11,60 |
| 25 | 38,17 | 17,13 |
| 26 | 25,90 | 15,37 |
| 27 | 28,80 | 12,79 |
| 28 | NV | NV |
| 29 | NV | NV |
| 30 | 21,44 | 11,54 |
| 31 | 20,95 | NV |
| 32 | 22,79 | 10,39 |
| 33 | 54,39 | 28,32 |
| 34 | 21,38 | 12,51 |
| 35 | 75,65 | 64,79 |
| 36 | 65,78 | NV |
| 37 | 78,36 | 38,17 |
| 38 | 78,89 | 80,66 |
| 39 | 21,06 | NV |
| 40 | 22,95 | NV |
| 41 | 30,16 | 13,03 |
| 42 | NV | NV |
| 43 | NV | NV |
| 44 | 39,97 | 12,42 |
| 45 | 49,94 | 14,55 |

Abbreviations: WB: Western Blot; Dys 1: dystrophin Rod domain antibody; Dys 2: dystrophin C-terminus antibody.
