# Supplementary figures and images for: Muscle histological changes in a large cohort of patients affected with Becker muscular dystrophy
Source: Acta Neuropathol Commun. 2022 Apr 8;10:48. doi: 10.1186/s40478-022-01354-3 (PMC8994373; doi:10.1186/s40478-022-01354-3)

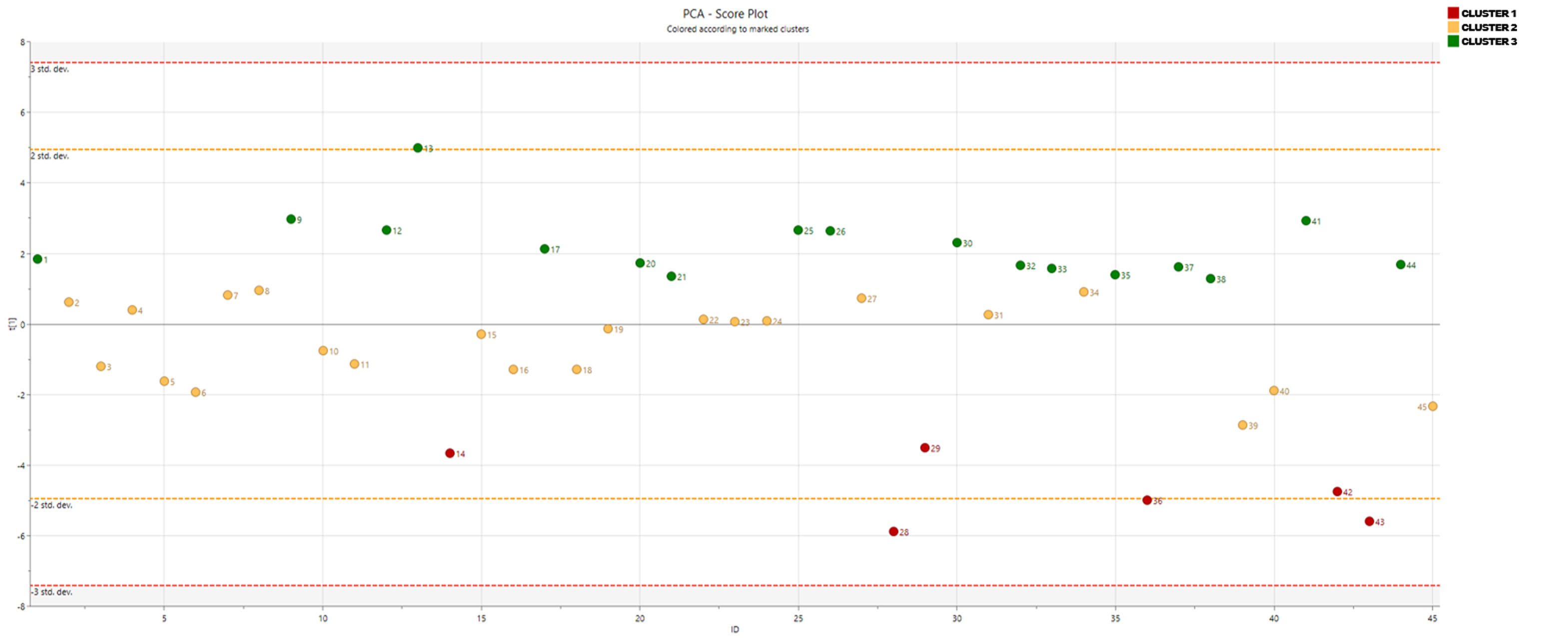

Supplement: Supplementary file 2 — Additional file 2 Bidimensional score plot of the principal component analysis (PCA) applied to the data set of the 45 BMD patients (each dot represents a patient) [file 40478_2022_1354_MOESM2_ESM.jpg]

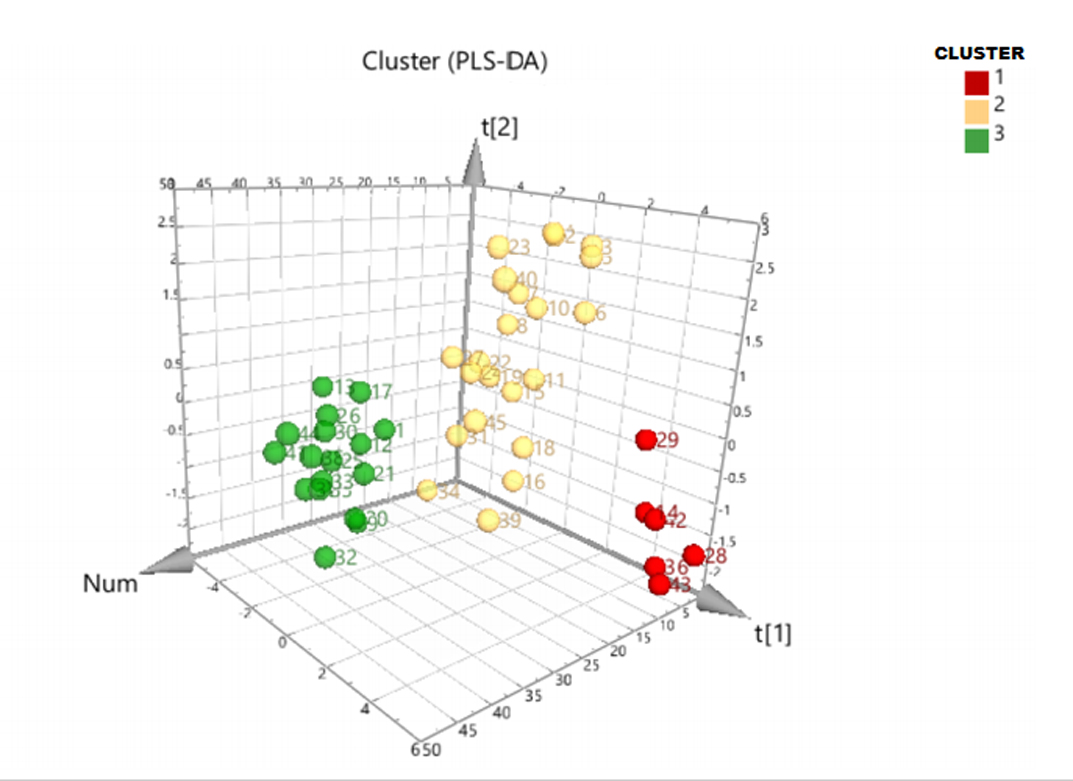

Supplement: Supplementary file 3 — Additional file 3 Three-dimensional score plot of the PLS-DA model showing the three clusters in which BMD patients are classified according to different histological and clinical traits. Each dot represents a patient. Green dots identified patients of cluster 1, blue dots of cluster 2 and red dots of cluster 3.The axis score t[1] represents the latent variable of the model. The latent variable is a mathematical construct that ‘summarizes’ the variables registered in the study. PLS-DA: partial least squaresdiscriminant analysis. [file 40478_2022_1354_MOESM3_ESM.jpg]

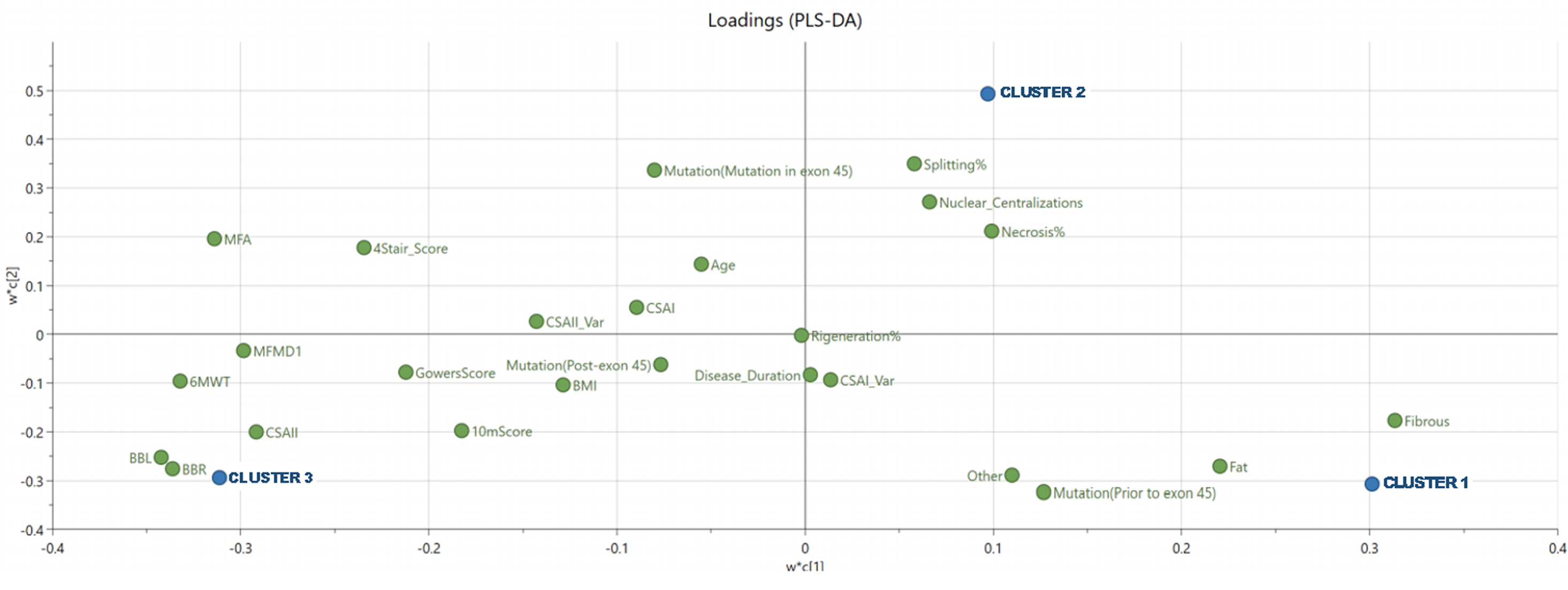

Supplement: Supplementary file 4 — Additional file 4 Loading plot of the PLS-DA model. The loading plot is complementary to the score plot and summarizes how the X-variables relate to each other as well as to group belonging (Y-variable symbolized by a group dot). X-variables located near a group dot are positively associated with that group. PLS-DA: partial least squares-discriminant analysis [file 40478_2022_1354_MOESM4_ESM.jpg]
